# Co-extraction of genomic DNA & total RNA from recalcitrant woody tissues for next-generation sequencing studies

**DOI:** 10.4155/fsoa-2018-0026

**Published:** 2018-04-25

**Authors:** Zhen Zeng, Tommaso Raffaello, Meng-Xia Liu, Fred O Asiegbu

**Affiliations:** 1 Department of Forest Sciences, University of Helsinki, Latokartanonkaari 7, 00014, Helsinki, Finland

**Keywords:** co-extraction, genomic DNA extraction, next-generation sequencing studies, total RNA extraction, wood samples

## Abstract

The successful implementation of next-generation sequencing techniques in plant and woody tissues depends on the quality of initial starting material. This study demonstrated the use of a modified protocol that enabled the simultaneous extraction of both genomic DNA and total RNA from recalcitrant woody material. The genetic material obtained by this protocol is of high quality and can be directly used in downstream analysis (e.g., next-generation sequencing). This protocol is particularly useful not only when the initial plant material is limited but also when genomic DNA features (e.g., methylation) have to be compared with the total RNA (e.g., gene expression). For such studies, the extraction from the same materials is highly preferred to minimize sample variation.

## Lay abstract

The advancement of next-generation sequencing techniques has greatly facilitated almost all branches of biological and life-science studies. To guarantee reliable results and conclusions, high-quality starting genetic material (genomic DNA and total RNA) is essential. Our modified protocol, which is based on existing methods, enabled the efficient simultaneous extraction of genomic DNA and total RNA from the same recalcitrant woody sample. Thereby, it greatly minimizes the amount of initial plant materials needed for a specific study.

The increasing affordability of next-generation sequencing (NGS) techniques such as RNA-seq and bisulfite sequencing has enabled the application of these techniques to a wide range of studies. The sequencing and the downstream analysis heavily rely on the quality of the starting biological materials and the efficacy of the extraction methods of the nucleic acids. Usually the genomic DNA (gDNA) and total RNA (totRNA) are extracted using separated batches of the same biological material. Few efforts have been made to combine the existing methods to simultaneously extract high-quality gDNA and totRNA from the same sample. This is especially important in the case of very limited amount of the starting material that cannot be split in different batches or in the case of studies where the analysis of gDNA and totRNA have to be compared so that the extraction from the same materials is highly preferred to minimize sample variation (for example, in DNA methylation studies) [1]. Protocols using TRIzol have been used for simultaneous isolation of DNA, RNA and proteins from cell and tissue samples [2]. However, compared with other chemicals, this reagent is more expensive and more toxic due to the phenol content. Moreover, the use of hazardous chemicals like phenol requires additional precautions in handling and disposal of the waste. Starting from already established protocols [3,4], we combined and developed a safe and cost-effective protocol for simultaneous extraction of gDNA and totRNA that can be applied to difficult and recalcitrant woody material. The resulting gDNA and totRNA have high quality and can be safely used for NGS studies.

The material used was obtained from a 30-year-old Norway spruce (*Picea abies* L. Karst.) tree inoculated with the fungal pathogen *Heterobasidion parviporum* Niemelä & Korhonen isolate 96026 in a forest of Lapinjärvi, Finland (60.5967 N, 26.1517 E) owned by Natural Resources Institute, Finland (Luke). The tree stems were drilled creating 15 mm diameter holes. The drilled holes were filled with Norway spruce sawdust precolonized by *H. parviporum* and sealed with parafilm. After three-and-half months, the inoculated stem tissues of 12 trees (three biological replicates N1, N2, and N3 with each replicate consisting of four pooled trees) were harvested in November. The surrounding area of the inoculated drill holes containing the mixture of tree woody tissues and fungal mycelia were collected and ground to powder by IKA^®^ A11 basic mill (IKA-Werke GmbH & Co. KG, Germany) in liquid nitrogen. The ground materials were then collected in 50 ml falcon tubes (2.0 g/tube). The step-by-step procedure for co-extraction of gDNA and totRNA is provided as a supplementary file (Supplementary Protocol). The gDNA concentration and integrity was determined by Qubit fluorimeter quantification and gel electrophoresis (1% Tris-acetate-EDTA agarose gel for 40 min at 150 V), respectively. The totRNA quality was assessed by Agilent 2100 bioanalyzer following the manufacturer's instructions.

Four extractions for each biological replicate were performed and pulled together to recover both the gDNA and the totRNA. A final total volume of 500 μl of gDNA and 320 μl of totRNA was obtained for each biological replicate. The concentration for gDNA was between 31.4 ng/μl and 276 ng/μl, while for totRNA was between 81 ng/μl and 772 ng/μl (Table 1). The protocol could extract the nucleic acids in a consistent way since, the amount of totRNA was always 2.2–2.8 times the amount of gDNA for each sample. The different amount of extracted nucleic acids from the same amount of starting wood material (2 g × 4 reactions) could reflect different level of genetic material available in the sample itself. The gel image indicated that the gDNA was of very high molecular weight with no appreciable degradation (Figure 1A). Additionally, the intensity of the gDNA bands was consistent with the Qubit fluorimeter quantification. The quality of the totRNA was also very high as indicated by the RNA integrity number (RIN), which was between 7.8 and 8.5 (Table 1 & Figure 1B–D). The totRNA quality could also be visually assessed by the ribosomal RNA peaks of 28S and 18S which showed a ribosomal RNA ratio in their intensity (28S/18S) between 1.9 and 2.9 (Figure 1B–D). The quantity and quality of the gDNA and totRNA extracted with this protocol have passed the sample quality control of Beijing Genomic Institute (www.genomics.cn/en/index) for bisulfite sequencing and RNA-seq, respectively. All submitted samples have reached the quality standard of Level A in Beijing Genomic Institute for Whole genome bisulfite sequencing and HiSeq Transcriptome library construction, respectively.

**Figure 1.**
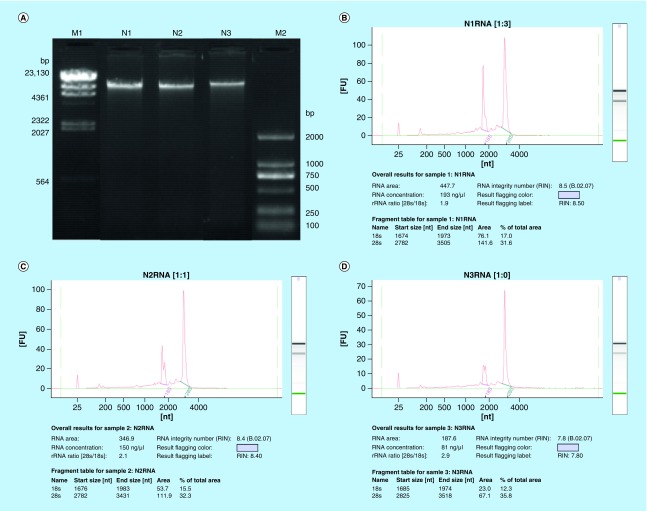
**Quality control of gDNA and totRNA.** **(A)** Electrophoretogram of gDNA. Lane M1 represents marker λ-Hind III digest (Takara). Lane M2 stands for marker D2000 (Tiangen). Lane N1, N2 and N3 represent gDNA samples. **(B)**, **(C)**, **(D)** Agilent profiles of N1, N2, N3 totRNA samples, respectively.

**Table 1.** **The concentration and quality of the extracted samples.**

**Genomic DNA**	**Concentration of DNA (ng/μl)**	**Total RNA**	**Concentration of RNA (ng/μl)**	**RIN**	**28S/18S**
N1	276	N1	772	8.5	1.9

N2	138	N2	300	8.4	2.1

N3	31.4	N3	81	7.8	2.9

RIN: RNA integrity number.

## Conclusion

With this paper, we provide the scientific community with an efficient, fast, safe and cheap protocol for simultaneous extraction of gDNA and totRNA from very difficult and recalcitrant samples like woody tissues. This protocol allows the extraction of high-quality genetic material for example, for whole-genome bisulfite sequencing and RNA-seq studies. The simultaneous extraction of both gDNA and totRNA can be an advantage in the case of limited source material or to minimize sample variation when the gDNA has to be compared with the totRNA from the same biological source or condition.

## Future perspective

NGS has and will continue to revolutionize the study of biology. The costs of sequencing are decreasing dramatically. To keep pace with increasing sequencing possibilities, upstream biological material preparations are heading toward more efficient and simplified directions. Our protocol contributes to that aspect and shall be readily applied in NGS studies.

**Executive summary**

- Our protocol is based on established methods for extracting genomic DNA and total RNA, separately.
- It allows simultaneous extraction of genomic DNA and total RNA from very difficult and recalcitrant samples like wood material.
- The resulting genetic materials are of high quality and could be easily applied in next-generation sequencing studies.

## References

[1] Zhu YJ, Xu J, Sun C (2015). Chromosome-level genome map provides insights into diverse defense mechanisms in the medicinal fungus *Ganoderma sinense*. *Sci. Rep.*.

[2] Chomczynski P (1993). A reagent for the single-step simultaneous isolation of RNA, DNA and proteins from cell and tissue samples. *Biotechniques*.

[3] Chang S (1993). A simple and efficient method for isolating RNA from pine trees. *Plant Mol. Biol. Rep.*.

[4] Fulton TM, Chunwongse J, Tanksley SD (1995). Microprep protocol for extraction of DNA from tomato and other herbaceous plants. *Plant Mol. Biol. Rep.*.

