# Supplementary material for: Co-extraction of genomic DNA & total RNA from recalcitrant woody tissues for next-generation sequencing studies
Source: Future Sci OA. 2018 Apr 25;4(6):FSO309. doi: 10.4155/fsoa-2018-0026 (PMC6060392; doi:10.4155/fsoa-2018-0026)
Supplement: Supplementary file 1 [file fsoa-04-306-s1.doc]

**PROTOCOL FOR:**

**Co-extraction of genomic DNA and total RNA from recalcitrant woody tissues for next-generation sequencing (NGS) studies**

Zhen Zeng, Tommaso Raffaello, Meng-Xia Liu, Fred O. Asiegbu

Department of Forest Sciences, University of Helsinki, Helsinki, Finland

## **REAGENTS**

Extraction buffer

SSTE buffer

1 M dithiothreitol (DTT) (Sigma-Aldrich)

Chloroform: isoamyl alcohol (24:1)

10 M LiCl

3 M NaAc

Isopropanol (Sigma-Aldrich)

70 % ethanol

Absolute ethanol (>=99.5%, Altia Oyj, Finland)

RNase A, DNase and protease-free (10 mg/mL, ThermoFisher Scientific)

Nuclease free water (Gibco™, Fisher Scientific)

Diethyl pyrocarbonate (DEPC)-treated water

MilliQ water

## **PROCEDURES**

**Day 1 for both genomic DNA (gDNA) and total RNA (totRNA) extraction**

1. Grind wood samples to fine powder by IKA^®^ A11 basic mill (IKA-Werke GmbH & Co. KG, Germany) with the presence of liquid nitrogen. Transfer the ground sample to a sterile 50 ml falcon tube.

*Note: make sure that the sample was ground to very fine powder and was frozen before adding the extraction buffer.*

1. Add 15 ml of **Extraction buffer** (for 2-3 g of material) (preheated at 65°C) and 150 μl of **1 M** **dithiothreitol** (**DTT)** to the sample. Mix well (vortex) and incubate for 5 min at 65°C.
2. Add equal volume of **chloroform: isoamyl alcohol (24:1).** Shake vigorously and centrifuge at 10,000 g (at room temperature) for 15 min. Transfer the upper aqueous phase to a new sterile 50 ml falcon tube. Repeat this step.
3. Add 1/4 volume of **10 M LiCl** to the supernatant and mix well by inverting the tubes several times. Precipitate the totRNA overnight at 4°C.

**Day 2 for gDNA extraction**

1. Centrifuge the mixture from step 4 at 10,000g for 30 min at 4 ℃. . Pour out the supernatant and save it in a new 50 ml falcon tube.

*Note: the supernatant collected in this step contains mostly gDNA.*

1. Add 1/10 volume of **3 M NaAc** and 1 volume of **isopropanol** (at room temperature) to the supernatant and incubate 5 min at room temperature.
2. Centrifuge at 10,000 g for 30 min at 4 °C. Discard the supernatant, wash the pellet with 2 ml of **70 % ethanol**. Centrifuge at 10,000 g for 10 min at 4 °C. Discard the supernatant and dry the pellet (around 10 min in a laminar flow hood).
3. Re-suspend the pellet in 900 μl (or less) of **nuclease-free water** and transfer to a new 2 ml eppendorf tube. Add **RNase A** at 100 µg/ml final concentration and incubate at 37 °C for 1-2 hr.
4. Add equal volume of **chloroform: isoamyl alcool (24:1)** and mix by shaking. Centrifuge at 4000 g for 10 min (at room temp). Transfer the supernatant to a new 2 ml eppendorf tube.

*Note: If the supernatant is not clean, this step could be repeated.*

1. Repeat step 6-7.
2. Dissolve the pellet in **nuclease-free water**. The gDNA is ready to be use.

**Day 2 for totRNA extraction**

1. Continue from step 5, after pouring out the supernatant for gDNA extraction, dry the pellet left in the tubes briefly. Dissolve the pellet in 1 ml of **SSTE** (preheated at 65°C).

*Note: the pellet collected in this step contains the totRNA.*

1. Add equal volume of **chloroform: isoamyl alcohol (24:1)**. Shake vigorously and centrifuge at 10,000 g for 15 min (at room temperature). Transfer aqueous phase to a new 2 ml tube (max 500 μl). Add 3 volumes of **cold absolute ethanol**. Precipitate it at -80°C overnight.

**Day 3 for totRNA extraction**

1. Centrifuge at 10,000 g at 4°C for 30 min. Pipette out the supernatant and dry the pellet. Dissolve the pellet in **nucleases-free water**. The totRNA is ready to be use.

## **RECIPES**

**Extraction buffer (50 ml) (**freshly made for each use)

Components Volume Final conc.

CTAB (hexadecyltrimethylammonium bromide, Sigma-Aldrich) 1g 2 %

PVP (Polyvinylpyrrolidione K 30, Honeywell, Fluka™) 1g 2 %

1 M Tris-HCl (pH 8.0) 5 ml 100 mM

0.5 M EDTA (pH 8.0) 2.5 ml 25 mM

4 M NaCl 25 ml 2 M

DEPC-treated water 17.5 ml

**SSTE buffer (50 ml)**

Components Volume Final conc.

4 M NaCl 12.5 ml 1 M

5 % SDS 5 ml 0.5 %

1 M Tris-HCl (pH 8.0) 500 µl 10 mM

0.5 M EDTA (pH 8.0) 100 µl 1 mM

DEPC-treated water 31.9 ml

**Chloroform: isoamyl alcohol (24:1, 250 ml)**

Measure 240 ml of Chloroform (Biotech grade, >=99.8 %, Honeywell Burdick & Jackson) with a sterile graduated cylinder and mix it with 10 ml of isoamyl alcohol (Molecular Biology, Fisher BioReagents).

**10 M LiCl (100 ml)**

Dissolve 42.4 g of lithium chloride (LiCl, Sigma Aldrich) in 90 ml of DEPC-treated water. Adjust the volume to 100 ml with DEPC-treated water.

**3 M NaAc (100 ml, pH 5.2)**

Dissolve 24.61 g of sodium acetate (NaAc, Sigma Aldrich) in 80 ml of sterile MilliQ water. Adjust the pH to 5.2 with glacial acetic acid. Adjust the volume to 100 ml with sterile MilliQ water.

**70 % ethanol (50 ml)**

Measure 35 ml of absolute ethanol and mix with 15 ml of sterile MilliQ water.

## **EQUIPMENT**

IKA^®^ A11 basic mill (IKA-Werke GmbH & Co. KG, Germany)

Eppendorf™ Centrifuge 5810R

Eppendorf™ Centrifuge 5424R

Water batch (TW series, JULABO GmbH)

Vortex mixer (Fisher Scientific)
